# Laser‐Architected Shape‐Configurable Vertical Graphene Thermoacoustic Loudspeakers for 3D Acoustic Emission

**DOI:** 10.1002/advs.202522911

**Published:** 2026-01-21

**Authors:** TaeGyeong Lim, Se Young Lee, Dohyung Lee, Baek Heon Lim, Jeongbo Lee, Wooseok Song, Sun Sook Lee, Sungwoong Park, Jin Kim, Soonmin Yim, Ki‐Seok An, Saewon Kang

**Affiliations:** ^1^ Thin Film Materials Research Center Korea Research Institute of Chemical Technology Daejeon Republic of Korea; ^2^ Department of Materials Science and Engineering Hanbat National University Daejeon Republic of Korea

**Keywords:** laser patterning, loudspeaker, thermoacoustic, vertical graphene

## Abstract

Recent advances in thermoacoustic (TA) loudspeakers have focused on freeform, conformal audio systems that enable conformal integration, spatial sound control, and seamless transformation between 2D and 3D geometries. However, their practical use remains limited by low sound pressure levels (SPLs) and poor mechanical adaptability, primarily due to insufficient thermal dissipation and structural rigidity. Here, we report a high‐performance, shape‐configurable TA loudspeaker based on vertically aligned reduced graphene oxide (VrGO), fabricated through a dual‐laser process combining continuous‐wave (CW) CO_2_ laser reduction with pulsed laser patterning. The CW laser induces vertical microstructuring and graphitization, enhancing thermal transport and reducing the heat capacity per unit area, while the pulsed laser enables precise patterning into complex geometries such as kirigami and 3D prismoids. The VrGO loudspeakers exhibit superior SPL (85 dB at 10 kHz), excellent heat dissipation, and high resilience, maintaining over 75% SPL under 500% strain and stable output over 1000 cycles. The patterned VrGO structures enable omnidirectional emission, conformal attachment, and 2D–3D transformation. Notably, even thick VrGO films retain strong acoustic performance, outperforming conventional TA devices. This scalable, material‐efficient strategy offers tunable directivity, adaptability, and multifunctionality for next‐generation wearable audio devices, interactive electronics, and conformal human–machine interfaces.

## Graphical abstract

A dual‐laser‐architected vertical graphene film enables high‐performance, morphologically reconfigurable thermoacoustic loudspeakers.The vertically aligned graphene structures combine efficient heat dissipation, thickness‐independent SPL, and kirigami‐driven 3D transformations, offering shape‐programmable, stretchable, and omnidirectional acoustic systems for future wearable and human–machine interface applications.

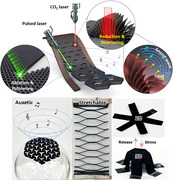

## 1 Introduction

Recent advances in interactive electronics have led to devices that are thin, flexible, and capable of arbitrary shape transformations, thereby enabling seamless integration with the human body, curved surfaces, and adaptive environments [1, 2, 3]. In particular, acoustic interface devices that can generate or detect sound have evolved beyond rigid, bulky speaker components toward film‐type sound sources that can bend, stretch, or dynamically morph for various futuristic applications, such as wearable electronics, immersive audio, augmented/virtual reality, soft robotics, and spatial sound systems [4, 5]. For this purpose, acoustic devices increasingly demand sound emitters that are lightweight, conformal, and functionally programmable, while maintaining consistent sound pressure levels (SPL) under mechanical deformation [6, 7]. Conventional electrodynamic loudspeakers are fundamentally constrained by their vibrating diaphragms, which limit their adaptability to soft or deformable platforms [8, 9, 10].

Thermoacoustic (TA) loudspeakers, which convert electrical energy into sound through the rapid Joule heating of a conductor, have attracted significant interest because they induce pressure oscillations in air via thermal expansion and offer a diaphragm‐free, solid‐state alternative well suited for flexible and conformable audio systems [11, 12, 13]. Despite these advantages, TA loudspeakers still exhibit lower energy efficiency than conventional loudspeakers, and their acoustic performance strongly depends on material thickness and thermal properties, which poses limitations to device design. Therefore, numerous studies have focused on developing TA loudspeakers by employing advanced nanomaterials and novel architectures over the past decade [14, 15, 16]. Various conductive nanomaterials, such as graphene, carbon nanotubes (CNTs), MXenes, and transition metal dichalcogenides, have been explored in TA loudspeakers to boost SPLs while maintaining mechanical flexibility [17, 18, 19, 20]. The ultralow heat capacity per unit area of nanometer‐thick CNT or graphene films enables a wide frequency response and high SPL in devices that are inherently lightweight, transparent, and stretchable [21]. 3D porous graphene structures, such as foams and aerogels, further improve performance by reducing heat leakage and increasing the surface area for heat exchange, allowing efficient thermoacoustic operation even with thicker films [22, 23, 24, 25]. These advances reveal an emerging materials design paradigm for TA loudspeakers that combines improved thermal management with flexible and stretchable architectures, paving the way for practical high‐performance TA devices.

In recent years, various patterning strategies, particularly kirigami‐based designs, have been explored to impart mechanical transformability and tunable spatial acoustic performance to TA loudspeakers [11]. By integrating strategic cuts into conductive films, these structures allow reversible transitions from flat to 3D geometries, enabling deformation into concave, convex, twisted, or stretched states without causing mechanical damage to the films. For example, kirigami‐based MXene TA speakers fabricated on ultrathin parylene substrates have demonstrated direction‐tunable sound radiation, omnidirectional SPLs, and stable output even under 50% strain [11, 26].

However, despite these advances, most kirigami‐structured TA devices suffer from intrinsic material limitations. Conventional planar films made from conductive nanomaterials, such as graphene, CNTs, and MXenes, exhibit a strong thickness dependence in SPL [11, 27]. As the film becomes thicker, heat cannot penetrate efficiently through the entire volume owing to limited thermal diffusivity, causing the inner regions of the film to remain acoustically inactive and thereby diminishing overall performance [23, 28, 29, 30]. To achieve high SPL, TA loudspeakers typically rely on ultrathin and mechanically fragile films [12, 31, 32, 33]. However, such structures inherently limit mechanical robustness, reduce power‐handling capability, and hinder scalability, thereby restricting their practical applicability in real‐world environments [33, 34].

To overcome these challenges, we introduce a scalable and simple fabrication route to realize high‐efficiency, shape‐configurable TA speakers using a continuous‐wave (CW) CO_2_ laser‐induced vertical graphene engineering. Our approach is based on a single‐step CO_2_ laser reduction process that locally transforms solution‐deposited graphene oxide (GO) films into a porous forest of out‐of‐plane reduced graphene oxide (rGO) walls, creating a high‐aspect‐ratio, vertically oriented morphology. This structure significantly enhances thermal diffusivity along the thickness direction and increases the surface area exposed to air, enabling efficient thermoacoustic conversion even in relatively thick films. Notably, our vertically aligned rGO (VrGO) loudspeakers demonstrate a significantly mitigated thickness dependence of SPL, breaking the traditional trade‐off between film thickness and performance [35, 36, 37, 38]. Furthermore, the pulsedlaser process simultaneously enables programmable kirigami patterning of VrGO films without requiring additional lithographic steps, providing a straightforward means to integrate material structuring and geometric configuration on a single platform. The resulting VrGO films are mechanically robust and compatible with kirigami patterning, allowing 2D‐to‐3D transformations into complex geometries such as cylindrical or dome‐like shapes. In these reconfigured states, the devices maintain stable acoustic output and exhibit omnidirectional sound radiation, demonstrating their potential as conformable, stretchable, and shape‐adaptive sound sources. By combining a novel material architecture with preprogrammed kirigami design, our devices establish a key platform for flexible acoustics and open new directions for wearable, spatial, and adaptive audio technologies.

## 2 Results and Discussion

**FIGURE 1:**
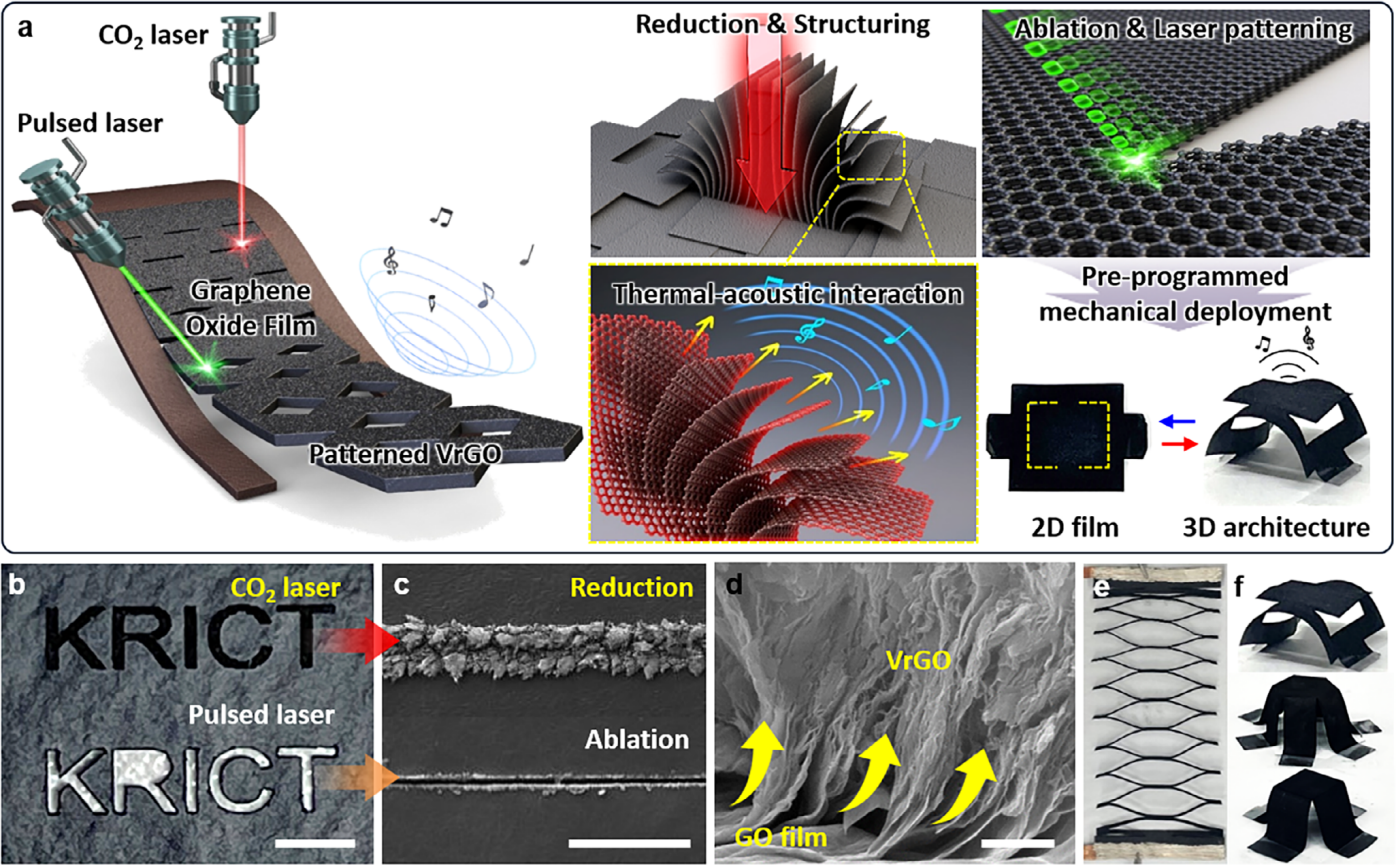
Fabrication, morphology, and structural features of patterned VrGO films using dual‐laser patterning. (a) Schematic illustration of the dual‐laser processing strategy for fabricating patterned VrGO TA loudspeakers and their working mechanism. (b,c) SEM images of CO_2_‐laser and pulsed‐laser‐irradiated GO films (scale bar: 500 µm). (d) SEM image showing vertically aligned rGO sheets in the VrGO structure (scale bar: 10 µm). (e) 100% stretched kirigami and (f) 3D‐structured VrGO films fabricated via pulsed laser‐based patterning.

Figure 1 illustrates a dual‐laser processing approach that combines material transformation and geometric patterning to produce high‐performance kirigami‐engineered TA loudspeakers. In this process, CW CO_2_ laser (λ = 10.6 µm) locally reduces GO films while simultaneously creating vertical microstructures through intense photothermal heating reaching up to ∼2500°C [39]. This process breaks C─O and C═O bonds, forming sp^2^ carbon networks, and generating VrGO walls with high specific surface area (Figure 1a). These vertically oriented architectures increase the air‐film contact, reduce the heat capacity per unit area (HCPUA), and improve the out‐of‐plane thermal transport for efficient thermoacoustic conversion. Together with this step, a pulsed laser (λ = 1.06 µm) is used in the same fabrication sequence to perform high‐resolution photothermal ablation by inducing highly localized heating with minimal heat diffusion to the surrounding area through its high repetition rate and short pulse duration. This enables the direct formation of precisely defined pattern cuts in the laser‐reduced VrGO regions. This combined process of the reduction and vertical alignment of graphene sheets with well‐designed patterning enables the fabrication of flat conductive films into shape‐configurable architectures capable of large, reversible 2D‐to‐3D shape changes and tunable acoustic directivity without compromising the electrical connection. Figure 1b presents the SEM images showing the distinct patterned regions created by the two different laser modes. The upper image displays the CO_2_‐laser‐treated VrGO region, where dense vertical graphene architectures walls rise uniformly from the film surface, forming a forest‐like microstructure with interconnected channels that increase the exposed surface area. The lower image shows the pulsed‐laser‐ablated pattern, where graphene oxide was clearly removed, producing sharply defined slit edges for the pattern cuts. Magnified SEM images show these contrasting morphologies more clearly, revealing VrGO walls with rough surfaces along the CO_2_‐laser‐treated area, whereas the pulsed‐laser‐ablated area exhibits precisely etched geometries with minimal thermal damage to the surrounding material (Figure 1c). Figure 1d shows a cross‐sectional SEM image of the VrGO structure formed by CO_2_ laser processing. The image clearly reveals the vertical alignment of the reduced graphene sheets, with microscale spacing extending above the compact rGO base layer, producing a hierarchical architecture. This structure provides efficient thermal pathways along the vertical direction while maintaining mechanical stability, enabling enhanced heat dissipation during the thermoacoustic operation. Meanwhile, ablation of the precisely using a pulsed laser facilitates the macrostructuring of the GO film into functional geometries such as kirigami, origami, and pop‐up prismoids (Figure 1e,f), thereby providing multifunctionality to the resulting TA devices. The resulting kirigami architecture can provide large uniaxial stretchability while maintaining electrical pathways, enabling a stable SPL output even under extreme deformation. Well‐defined laser patterning process enabled the transformation from 2D‐to‐3D structures, demonstrating the potential of VrGO‐based designs as freeform thermoacoustic emitters for spatial audio, interactive electronics, and conformal human–machine interfaces (Figure 1f).

**FIGURE 2:**
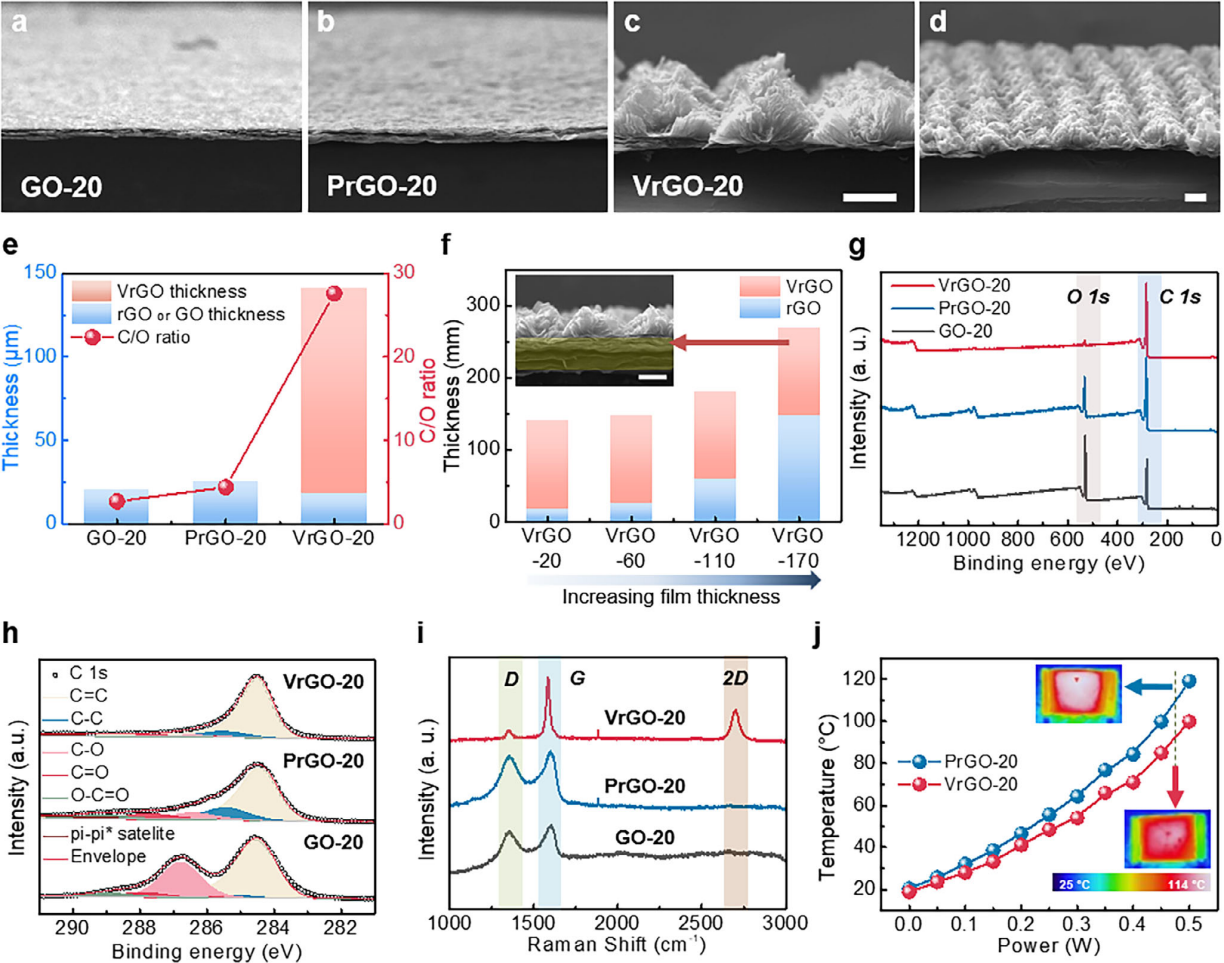
Structural, chemical, and thermal characteristics of GO, PrGO, and VrGO films. SEM images of (a) GO‐20, (b) PrGO‐20, and (c, d) VrGO‐20 films (scale bar: 100 µm). (e) Thickness comparison of GO‐20, PrGO‐20, and VrGO‐20 films. (f) Thickness comparison of VrGO films with varying initial GO film thicknesses. (g) XPS survey spectra, (h) deconvoluted C 1s spectra, and (i) Raman spectra of GO‐20, PrGO‐20, and VrGO‐20 films. (j) Surface temperature variations of PrGO‐20 and VrGO‐20 under Joule heating at different power levels.

The structural and chemical changes of the VrGO induced by CO_2_ laser treatment were systematically investigated in comparison to a planar reduced GO film (PrGO) obtained by conventional thermal annealing. Figure 2a,b show cross‐sectional SEM images of the PrGO film, confirming a mostly layered structure with a slight thickness increase to 25.1 µm (from an initial GO film thickness of 20.3 µm) due to thermal expansion. In clear difference, the CO_2_ laser‐treated film exhibits a two‐layer structure consisting of a dense PrGO base (≈17.8 µm thick) with an upper region of VrGO wall (Figure 2c,d). The vertically protruding rGO layers increase the total film thickness significantly to 140.9 µm (Figure 2d), about a six‐fold increase over the original film (Figure 2e). This significant increase is attributed to the photothermal effect of the CO_2_ laser, which rapidly heats the GO, causing outgassing and exfoliation, resulting in graphene sheets to align vertically [39, 40]. Figure 2e shows the thickness changes of the original GO film (20.3 µm) expanding to 25.1 µm after thermal reduction, whereas laser structuring yields a 17.8 µm PrGO base with 123.1 µm of VrGO (total 140.9 µm). Importantly, not all of the GO was converted to a vertical structure during the laser process, and the remaining PrGO layer served as a stable base. The formation of the VrGO layer appeared to be self‐limited to a certain thickness.

To further investigate the vertical architecture formation and the heat dissipation behavior of VrGO, we fabricated a series of VrGO films from GO bases of varying thickness (20, 60, 110, and 170 µm, named as GO‐20, GO‐60, GO‐110, and GO‐170) under identical laser conditions. The resulting laser‐reduced films (VrGO‐20, −60, −110, −170) were compared with the corresponding thermally annealed films (PrGO‐20, −60, −110, −170). The final thicknesses of the PrGO films were 25.1, 119.7, 167.4, and 261.4 µm for initial GO thicknesses of 20, 60, 110, and 170 µm, respectively (Figure S1) owing to thermal expansion upon thermal reduction. Meanwhile, the laser‐treated VrGO films were approximately 140.9, 147.6, 180.1, and 268.6 µm for GO‐20, GO‐60, GO‐110, and GO‐170, respectively (Figure S2), showing a noticeable increase in film thickness under laser‐assisted reduction compared to that of the PrGO films. Remarkably, in all VrGO films, the thickness of the VrGO region stayed almost constant at 121–123 µm, while the thickness of the PrGO base increased in proportion to the initial GO film thickness (Figure 2f)

X‐ray photoelectron spectroscopy (XPS) and Raman analyses were performed to compare the chemical compositions and graphitic orders of GO, PrGO, and VrGO (Figure 2g,h,i). The XPS survey spectra showed that the C/O atomic ratio increased from roughly 2.7 in GO to 4.4 in thermally reduced PrGO and further to ∼27.6 in laser‐structured VrGO, indicating a drastically lower oxygen content in the VrGO film (Figure 2g). The high C/O ratio of VrGO indicates a more complete removal of oxygen functional groups by intense laser reduction. Detailed XPS C 1s spectra showed that VrGO had a dominant sp^2^‐hybridized carbon peak and much smaller peaks for C─O, C═O, and O─C═O bonds than GO and PrGO (Figure 2h). Quantitative deconvolution of the C 1s spectrum confirmed that VrGO contained a much higher fraction of graphitic (C═C) bonds and far fewer oxygenated carbon species [39]. Raman spectroscopy further confirms the enhanced degree of graphitization of the laser‐reduced films (Figure 2i). The Raman spectrum of VrGO displays distinct D (∼1350 cm^−1^) and G (∼1585 cm^−1^) bands, along with a clear 2D band around 2700 cm^−1^. In contrast, the Raman spectra of GO and PrGO show broad and overlapping D/G bands with no clear 2D band, indicating a highly disordered structure. The presence of a strong 2D band and more defined D and G peaks in VrGO indicate that the laser‐induced VrGO layer has a highly graphitized and multilayered graphene structure, which cannot be achieved by thermal reduction alone. Consistent with its graphitic nature and 3D microarchitecture, VrGO exhibited better heat dissipation. Under the same Joule heating conditions, the surface temperature of the VrGO film was significantly lower than that of the PrGO film (Figure 2j). This improved thermal behavior of VrGO was attributed to the vertically aligned graphene network with numerous air gaps between the separated graphene sheets, which provided efficient through‐thickness heat pathways. Moreover, owing to the laser‐induced high‐temperature reduction process, the VrGO structure is expected to possess sufficient intrinsic thermal stability under operating temperatures exceeding 100 °C.

**FIGURE 3:**
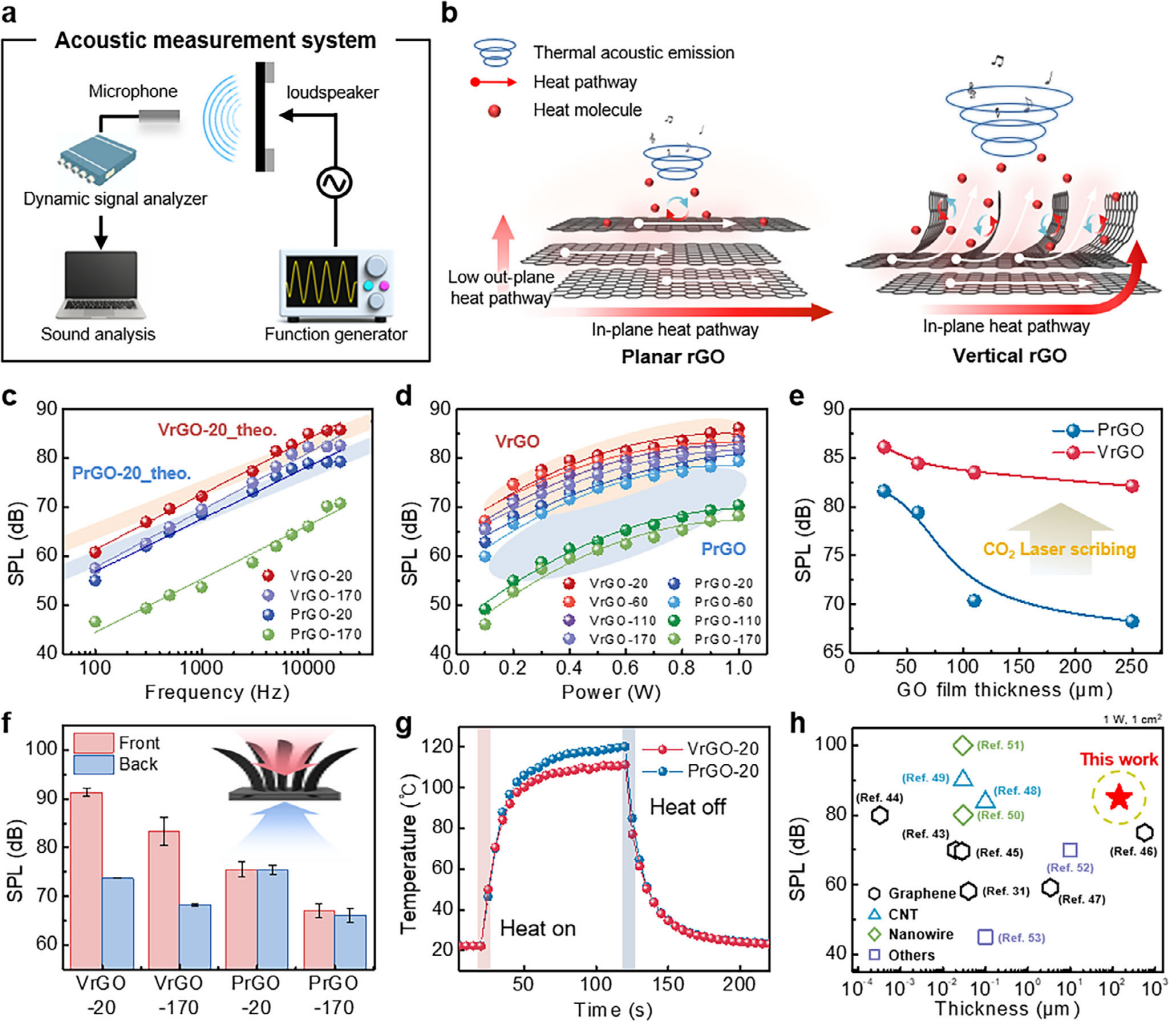
Acoustic and thermal performance of VrGO vs. PrGO TA loudspeakers. (a) Schematic illustration of the acoustic measurement setup inside the anechoic chamber. (b) Schematic comparison of heat dissipation properties between VrGO and PrGO films. (c) SPL spectra of VrGO and PrGO films over the 100 Hz–20 kHz frequency range. (d) SPL of VrGO and PrGO films at various input power levels. (e) Thickness‐dependent SPL comparison between VrGO and PrGO films. (f) Directional SPL measurements of VrGO and PrGO films from the front and back sides. (g) Temperature–time curves of VrGO‐20 and PrGO‐20 under Joule heating with an applied power of 0.5 W. (h) Comparison of the normalized SPL of the present TA loudspeaker with previously reported TA devices as a function of device thickness. All data are normalized to 1 cm^2^ active area and measured at 10 kHz under the same input power, highlighting the impact of device thickness on thermoacoustic performance.

This efficient heat management enables the VrGO films to provide superior TA loudspeaker performance. The acoustic performance of our TA loudspeakers was evaluated by applying AC voltages to the films and measuring the emitted sound using a calibrated microphone with analysis using a dynamic frequency analyzer (Figure 3a; Figure S3). All measurements were performed inside a custom‐built soundproof chamber to suppress ambient noise and ensure reliable results. In general, conventional PrGO loudspeakers suffer from inefficient out‐of‐plane heat dissipation owing to their layered 2D structure, which severely limits the thermal exchange with the surrounding air and leads to low SPL performance [23, 41, 42]. In contrast, the VrGO loudspeaker, with its superior graphitic order and microstructured 3D design, provides improved acoustic characteristics. To conceptually highlight these differences, we present a schematic comparison of PrGO and VrGO architectures (Figure 3b). The forest of out‐of‐plane graphene walls in the VrGO film is expected to provide a greatly increased air‐contact surface area and more efficient through‐thickness heat transfer, promoting rapid thermal exchange with air, thus generating highly improved TA performance.

To quantify the effect of this structural difference on the thermal properties, we measured the specific heat capacities of the films using laser flash analysis (LFA). Notably, the VrGO‐20 showed a specific heat of 0.503 J g^−1^ K^−1^, which is substantially lower than that of PrGO‐20 (0.718 J g^−1^ K^−1^). This ∼30% reduction in specific heat indicates that the VrGO film has a lower HCPUA, indicating that less energy is required to increase its temperature for efficient thermoacoustic sound generation. Figure 3c compares the sound pressure level (SPL) spectra of VrGO‐20 and PrGO‐20 measured across frequencies from 100 Hz to 20 kHz. The VrGO‐20 loudspeaker achieves an SPL of 85.0 dB at 10 kHz, significantly exceeding the PrGO‐20, which reaches only 78.9 dB at the same frequency (Figure 3c). The predicted SPL curves of VrGO‐20 and PrGO‐20 obtained from their measured heat capacities were consistent with the experimental results. The improved performance of the VrGO device can be explained in terms of classical thermoacoustic theory. According to a model based on Arnold and Crandall's formulation, (Equation 1) [23].

(1) $$P_{rms}=\frac{\sqrt{\alpha }\rho_{0}}{2\sqrt{\pi }T_{0}}\cdot \frac{1}{r}\cdot P_{input}\cdot \frac{\sqrt{f}}{C_{s}}\cdot$$

where *P_rms_* is the root‐mean‐square sound pressure, *r* is distance between the loudspeaker and microphone, *P_input_* is input power, *f* is the sound frequency, *C_s_* is heat capacity per unit area, and α, ρ_0_, and *T*_0_ is the thermal diffusivity, density, and temperature of the ambient gas, respectively. This indicates that lowering the heat capacity of the film using thinner films or materials with intrinsically lower specific heat is essential for achieving a high SPL in TA loudspeakers. Consistent with Equation (1), we observed that the SPL of our TA loudspeaker decreased with increasing distance from the sound source, following the expected 1/*r* attenuation in the sound pressure (Figure S5). This distance‐dependent behavior is consistent with theoretical predictions and further confirms the reliability of our measurement arrangement and analysis. Notably, our results show that the VrGO loudspeaker significantly overcomes the limitation of the conventional thickness. For example, even though the VrGO‐170 film is over eight times thicker than VrGO‐20, it still produced 82.6 dB at 10 kHz, indicating small reduction with only about 2.4 dB lower than the much thinner VrGO‐20 (85.0 dB). By contrast, when the PrGO film's thickness was increased to 170 µm, its SPL at 10 kHz plummeted to 66.1 dB, a drop of nearly 13 dB compared to PrGO‐20 (78.9 dB). These results show that the vertical graphene structure effectively preserves the acoustic performance even in relatively thick films, whereas conventional stacked graphene structures rapidly lose their efficiency as the thickness increases. Figure 3d further highlights this behavior by plotting the SPL at 10 kHz as a function of the input power for VrGO and PrGO films of various thicknesses. In all cases, the SPL increased approximately linearly with the input power, and at a given power, the VrGO films consistently produced a higher SPL than the PrGO films. Importantly, as the film thickness increased, the SPL of the PrGO devices deteriorated sharply, whereas the VrGO devices maintained high performance with only a slight reduction (Figure 3e). This pattern demonstrates the effectiveness of the vertically aligned graphene network in facilitating the out‐of‐plane heat dissipation, which reduces the usual negative effect of increasing the thickness of the underlying planar rGO layer (Figure 3e).

We also performed directional sound measurements to verify the specific contributions of the VrGO layers. For each freestanding loudspeaker, SPL was recorded from the front (VrGO side) and back (PrGO side) of the film (Figure 3f). While the PrGO film emitted the same SPL in both directions, the VrGO films showed a clear directional difference, with the side containing the laser‐induced vertical graphene layer producing a much higher SPL than the opposite side. For instance, both VrGO‐20 and VrGO‐170 yielded significantly stronger sounds when the VrGO face was oriented toward the microphone, confirming that the majority of the acoustic emission originated from the vertically structured graphene layer (Figure 3f). In addition, we compared the thermal responses of the VrGO and PrGO films under Joule heating to assess their heat‐dissipation capabilities. The surface temperature of each film was monitored by applying a fixed electrical power and then turning off the power (Figure 3g). The VrGO‐20 film consistently exhibited a lower steady‐state temperature than PrGO‐20 under the same heating conditions and cooled much faster once the heating was turned off. This faster thermal relaxation demonstrates the superior heat dissipation of the VrGO design because the porous vertical graphene network efficiently transferred heat to the surrounding air (Figure 3g). Consequently, the VrGO‐based TA loudspeakers exhibited remarkable acoustic performance. When compared at a normalized input power, the VrGO films demonstrate superior acoustic efficiency considering their substantial thickness, achieving results competitive with previous reports (Figure 3h; Table S1) [31, 43, 44, 45, 46, 47, 48, 49, 50, 51, 52, 53].

**FIGURE 4:**
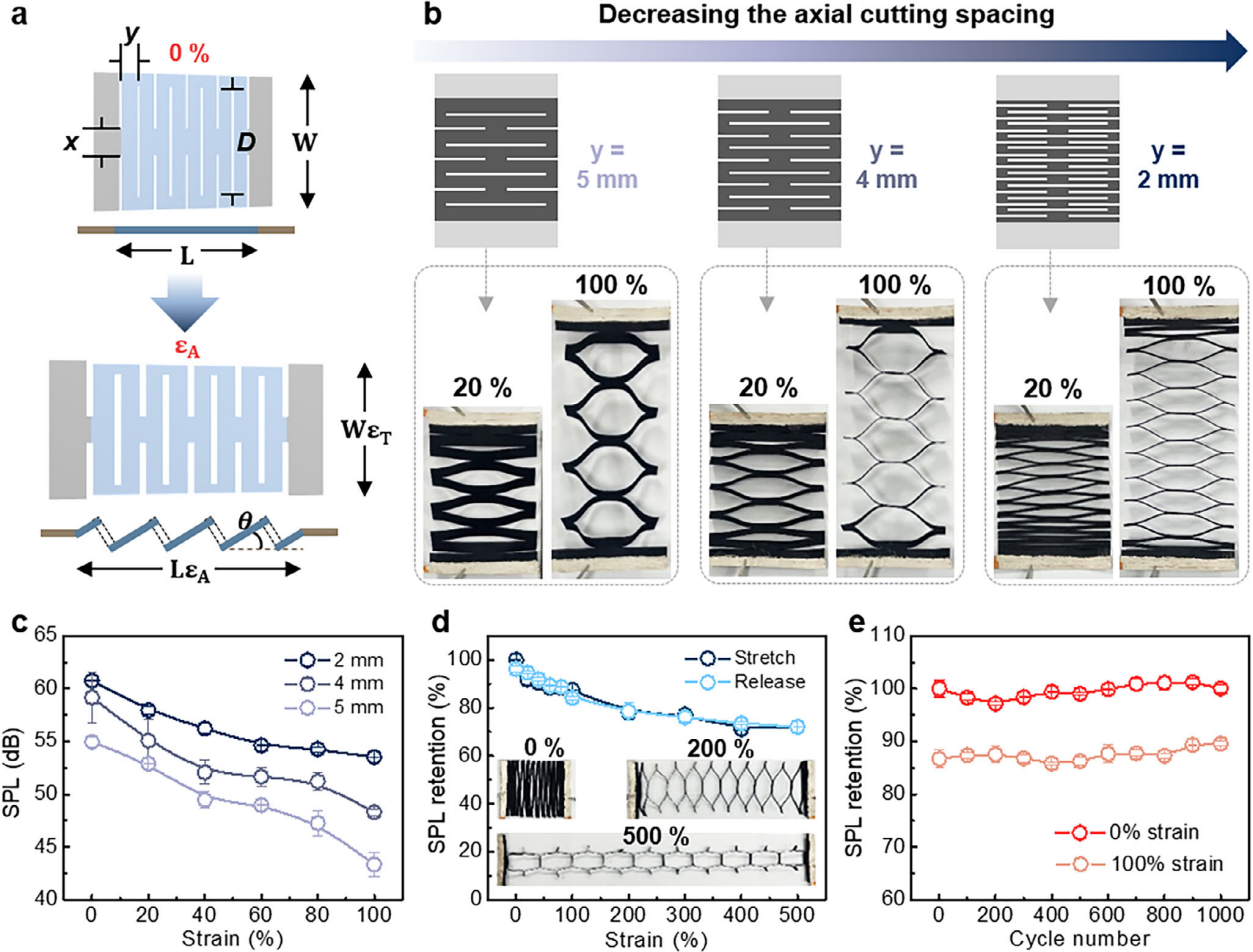
Stretchable kirigami‐patterned VrGO TA loudspeakers. (a) Geometry of the kirigami structure. (b) Digital images of kirigami‐patterned VrGO TA loudspeakers with different axial cut spacings under mechanical strain. (c) SPL retention of kirigami‐patterned VrGO TA loudspeakers with varying axial cut spacing as a function of strain. (d) SPL retention of kirigami‐patterned VrGO TA loudspeakers with an axial cut spacing of 2 mm under up to 500% strain. (e) Durability over 1000 repeated stretching‐releasing cycles.

The 3D architecture of VrGO serves as a promising platform for various mechanically functional patterning approaches, enabling TA loudspeakers with stretchable and freeform designs and tunable acoustic performances. For this purpose, various structurally patterned VrGO‐based TA loudspeakers were fabricated using pulsed laser‐based patterning techniques. Patterned VrGO TA loudspeakers were produced by applying pulsed laser cutting to GO films, followed by CW CO_2_ laser reduction on both sides of the GO films. The resulting patterned VrGO TA loudspeakers contained VrGO on both sides of the GO films, as shown in Figure S6. Figure 4a schematically illustrates the VrGO loudspeaker patterned with a 1D kirigami lattice, highlighting that axial stretching is transformed into rotation and bending at the cut hinges with minimal transverse contraction [54, 55]. The array of parallel slits acts as a mechanical hinge, greatly amplifying the deformability. This kirigami‐engineered mechanical geometry allows stiff and planar films to stretch like springs, converting in‐plane tension into rotations rather than bulk material strain [54, 55, 56]. Theoretical analysis of this geometry is presented in Supplementary Note 1, and confirms that the mechanical deformability and deformation modes of the kirigami patterned structure are governed by the cut geometry [55, 57]. To investigate the effect of the pattern geometry, Figure 4b compares kirigami‐VrGO films with different axial cut spacings y (the distance between adjacent slits along the stretch direction) of 5, 4, and 2 mm. The kirigami‐patterned VrGO structures were successfully fabricated in a substrate‐free configuration across a wide range of slit widths, and they exhibited excellent stretchability and mechanical stability under various applied strain levels from 0 to 100% (Figure S7). Among them, kirigami‐structured VrGO with denser cutting, which is a smaller y value, appears to exhibit more stable stretchability (Figure S7), which can be explained by the theoretical analysis in Supplementary Note 1. As illustrated in the geometric model, the maximum attainable rotation angle (θ_*MAX*_) of the kirigami features is solely determined by the cut geometry [58]. A reduction in y value increases θ_*MAX*_, thereby raising the upper limit of axial strain that the structure can accommodate and enhancing its maximum stretchability. These observations simply demonstrate that the cut spacing can be tuned to preprogram the deformability of the device. This tunable stretchability is particularly beneficial for wearable applications, enabling the integration of a more compliant loudspeaker into 3D architectures. To achieve this, the performance stability of the SPL under large deformations enabled by the kirigami patterns should be carefully considered. Figure 4c shows the SPL retention as a function of the axial strain for three cut spacings of 5, 4, and 2 mm. The TA speaker with denser cuts maintained a greater level of sound output under stretching. For instance, at 100% elongation, the kirigami TA loudspeaker with a cut spacing of 2 mm preserved most of its original SPL, whereas that with a cut spacing of 5 mm showed a more noticeable drop (Figure S8). The 2 mm design maintains a high SPL even as the strain increases because many small cut hinges distribute the deformation. These results highlight that the kirigami approach not only enables deformability but also crucially protects the acoustic performance stability by structural strain management. To further explore the limits of deformability, the 2 mm kirigami VrGO loudspeaker endured extreme uniaxial stretching up to 500% strain while maintaining a strong acoustic output (Figure 4d). When the kirigami structure expands into a wide mesh, the interconnected hinge strips preserve conductive pathways and evenly distribute deformation, preventing failure and enabling robust TA performance, retaining up to 75% of its original SPL even under a uniaxial strain of 500%. Owing to the kirigami geometry characteristics and mechanical durability of VrGO film, kirigami VrGO loudspeaker with the smallest cut spacing of 2 mm yields the highest mechanical deformability and prevented stiffening, allowing the device to withstand stretch‐release cycle at strains exceeding 500% without noticeable degradation in acoustic performance. Figure 4e shows the cycling durability of the 2 mm kirigami‐patterned VrGO loudspeaker by plotting its SPL retention over 1000 stretch–release cycles at 100% strain. The loudspeaker exhibited outstanding long‐term mechanical durability as the SPL remained high and nearly constant throughout the cyclic test, with no significant degradation after thousand stretching cycles (Figure 4e; Figure S9). Notably, the TA performance measurements conducted during the mechanical cycling inherently involved repeated thermal cycling, suggesting stable acoustic performance under combined mechanical and thermal cycling conditions. To further examine potential structural or bonding changes after repeated operation, we conducted post‐cycling characterization using SEM and Raman spectroscopy. SEM images confirmed that the periodic vertically aligned VrGO architecture and the spacing between adjacent vertical graphene sheets were well preserved after cyclic operation (Figure S10). Raman spectra showed a slight increase in the D‐band intensity, while maintaining an overall similar spectral profile, indicating only minimal defect evolution without significant structural degradation (Figure S11).

In addition, it should be noted that kirigami‐based patterning inherently involves geometry‐dependent trade‐offs between mechanical deformability and the continuity of thermal and electrical transport pathways. At higher pattern densities or under more extreme deformation conditions, such patterning can weaken the structural integrity and disrupt the continuous pathways necessary for efficient heat and current flow, potentially affecting device durability and sound quality of TA loudspeakers that rely on Joule heating–induced sound pressure generation.

In this respect, the stable mechanical and acoustic performance of the 2 mm kirigami‐patterned VrGO loudspeaker suggests that the applied kirigami pattern density remains compatible with efficient thermoacoustic operation under the deformation conditions explored in this work.

**FIGURE 5:**
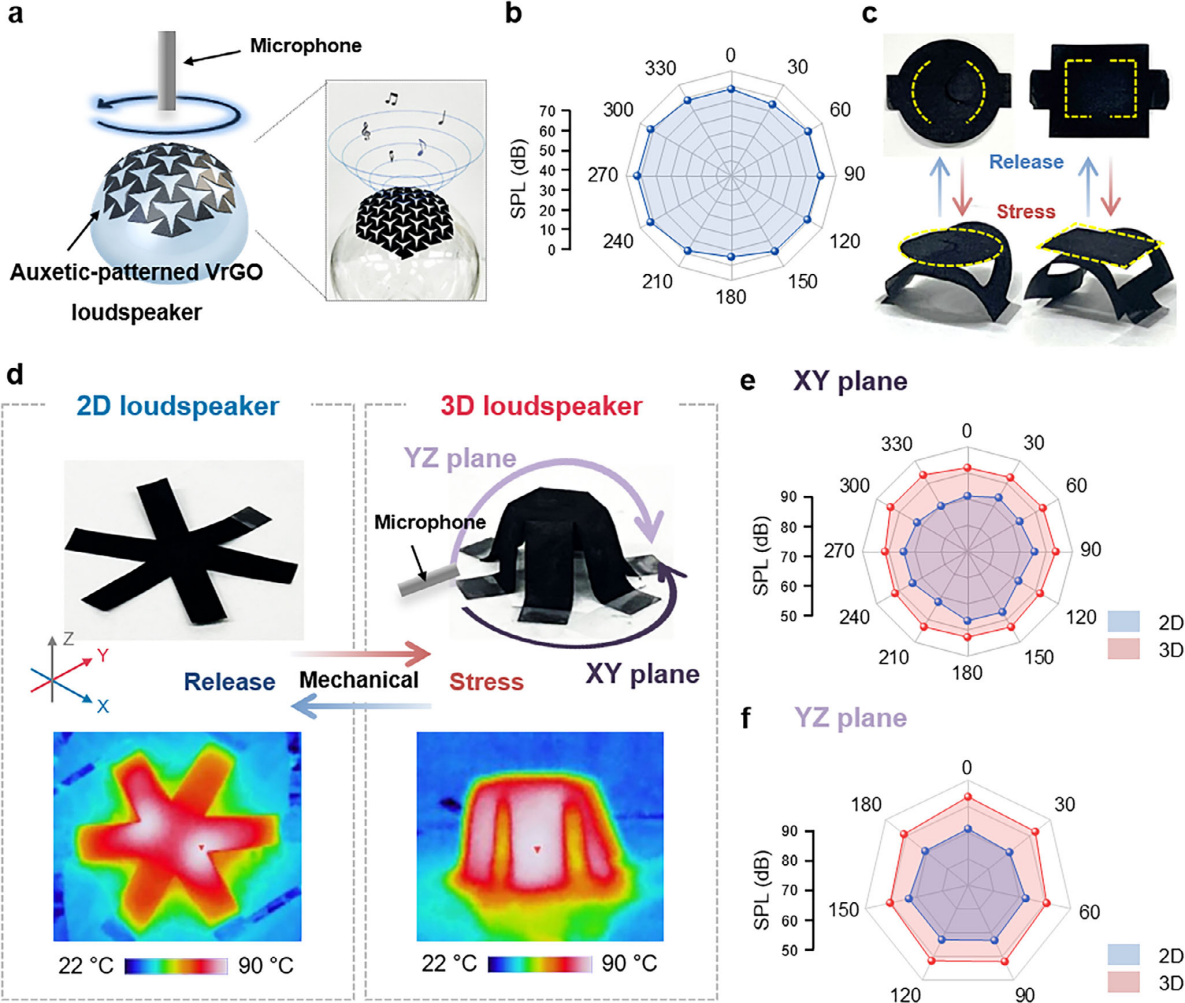
Multifunctional and shape‐configurable VrGO TA loudspeakers. (a) Auxetic‐patterned VrGO TA loudspeaker for conformal surface attachment. (b) SPL of the auxetic‐patterned VrGO TA loudspeaker on curved surfaces over 360°. (c) 3D‐transformable VrGO film. (d) Digital images and infrared (IR) camera images of the hexagonal prismoid‐shaped loudspeaker. (e, f) SPL maps of the hexagonal prismoid‐shaped loudspeaker in 2D and 3D configurations across the XY and YZ planes.

Such mechanical functionality with huge deformation greatly expands the potential applications, allowing the integration of loudspeakers into form factors that undergo large shape changes, such as inflatable or morphing structures, without loss of sound output. We further demonstrated mechanical multifunctionalities by engineering various structural patterns of VrGO TA loudspeakers (Figure 5). An auxetic‐patterned VrGO TA loudspeaker was introduced for conformal attachment to curved surfaces (Figure 5a). Auxetic geometry, characterized by a negative effective Poisson's ratio, allows the structure to expand uniformly in both the axial and transverse directions under tension [59, 60, 61]. This strain redistribution minimizes localized stress, prevents delamination on curved substrates, and preserves conductive continuity. Consequently, the auxetic‐patterned VrGO loudspeaker exhibited spatially uniform SPLs when mounted on convex geometries, confirming its ability to achieve omnidirectional sound emission with minimal distortions (Figure 5b; Figure S12). In addition to conformability, patterned VrGO films with various prismoid patterns and pop‐up structural design were prepared for 2D‐to‐3D transformations, enabling structural reconfiguration and functional enhancement (Figure 5c; Figure S13). A hexagonal prismoid VrGO TA loudspeaker was fabricated to enhance acoustic radiation in three dimensions (Figure 5d; Figure S14 and Supporting video S1). This architecture showed a uniform surface temperature distribution under Joule heating in both flat and folded states, confirming consistent thermal actuation across the configurations (Figure 5d; Figure S15). Acoustic measurements in the XY and YZ planes demonstrated that the 3D prismoid configuration provided significantly more spatially stable SPLs than the 2D form (Figure 5e, f). This improvement arises from the geometric advantage of the prismoid, which increases the effective radiating area, improves the coupling with the surrounding air, and distributes heat sources more evenly in space, thereby enhancing omnidirectionality. Similarly, a rectangular prismoid VrGO loudspeaker was developed, which exhibited stable thermal behavior and spatially uniform acoustic radiation (Figure S16). This integration with the VrGO film and kirigami engineering with geometric patterning not only enables conformability but also provides tunable acoustic directivity and improved spatial uniformity, capable of providing a futuristic platform of pre‐programmed, free‐formed loudspeakers.

## 3 Conclusion

In this study, we demonstrated a facile and scalable strategy for fabricating high‐performance, shape‐configurable TA loudspeakers based on VrGO films. By integrating a dual‐laser processing approach combining CW CO_2_ laser irradiation with pulsed laser patterning, we simultaneously achieved localized reduction, vertical microstructuring, and geometric programmability within a facile process. The VrGO films obtained via CO_2_ laser irradiation exhibited hierarchical 3D graphitic architectures with efficient thermal dissipation and mechanical robustness. Compared with conventional PrGO films, the VrGO‐based TA loudspeakers exhibited significantly higher SPLs over a broad frequency range and maintained relatively stable acoustic performance even with increased film thickness. Moreover, various patterned VrGO thermoacoustic loudspeakers were fabricated using fine pulsed laser cutting within a defined range of pattern densities, while retaining stable mechanical integrity and effective thermal and electrical transport for robust acoustic performance. The kirigami‐engineered VrGO architectures enabled stretchable loudspeakers that retained their SPL under extreme strains up to 500% and demonstrated excellent durability over repeated mechanical cycling. In addition, various structural designs were realized, such as auxetic, hexagonal, and rectangular prismoid patterns, expanding the functionality of VrGO films, allowing conformal attachment to curved surfaces and omnidirectional sound generation through 3D transformations. These laser‐processed kirigami structures exhibit uniform surface heating and well‐distributed SPL, highlighting VrGO as a versatile platform for next‐generation wearable and shape‐adaptive acoustic devices. Further exploration of diverse kirigami geometries and structural configurations will enable enhanced acoustic performance and device adaptability.

## 4 Experimental Section

### 4.1 Preparation of VrGO and PrGO Films

Freestanding GO films with different thicknesses were prepared by vacuum filtration of a commercial GO solution (1 wt%, Nano GO, Grapheneall, South Korea) at loadings of 40, 80, 160, and 400 mg through an anodic aluminum oxide (AAO) membrane filter (0.2 µm pore size, 47 mm diameter, Anodisc 47, Cytiva, United Kingdom). The resulting samples are denoted as GO‐20, GO‐60, GO‐110, and GO‐180, respectively. The as‐prepared GO films were dried overnight under vacuum at room temperature prior to further processing. Film thicknesses were confirmed using cross‐sectional SEM imaging. Subsequently, the films were subjected to direct laser irradiation using a CW CO_2_ laser (λ = 10.6 µm, 40 W, C30, Koreart) with a scanning speed of 200 mm s^−1^, a line density of 5 lines mm^−1^, and a laser power of 6.8 W. The laser‐irradiated GO films were then thermally annealed in a tube furnace (Lindberg/Blue M, Thermo Scientific, USA) at 200 °C for 1 h under a continuous Ar flow (99.999% purity, 200 sccm). The furnace ramping rate was 5 °C min^−1^, and the samples were naturally cooled to room temperature. This process ensured uniform thermal reduction and the removal of residual oxygen functionalities, yielding VrGO films. The resulting samples from the precursor GO‐20, GO‐60, GO‐110, and GO‐180 films were labeled VrGO‐20, VrGO‐60, VrGO‐110, and VrGO‐180, respectively. For comparison, PrGO films of corresponding thicknesses were obtained by subjecting pristine GO‐20, GO‐60, GO‐110, and GO‐180 films to the same thermal annealing protocol without prior laser irradiation.

### 4.2 Laser Patterning of VrGO Films

VrGO films were patterned using a nanosecond pulsed fiber laser system (λ = 1.06 µm, 30 W, K9 fiber laser, i‐Jet Korea) operated at a repetition frequency of 80 kHz. Laser processing was conducted with a scanning speed of 500 mm s^−1^, a line density of 20 lines mm^−1^, and a laser power of 24 W under ambient conditions. Kirigami and auxetic patterns were designed using CAD software (AutoCAD 2024, Autodesk, USA) and exported as DXF/DWG files, which were directly imported into the laser control software for high‐precision ablation. These parameters were optimized to achieve high‐resolution and reproducible kirigami architectures while minimizing the collateral thermal damage.

### 4.3 Characterization

The morphologies and microstructures of the samples were examined using scanning electron microscopy (SEM, Vega II LSU, TESCAN, Czech Republic). Raman spectroscopy (XperRam35V, Nanobase, λ = 532 nm) and XPS (K‐Alpha, Thermo Fisher Scientific, USA; Al Kα radiation, hν = 1486.6 eV) were employed to analyze the chemical bonding states and structural changes. The XPS spectra were deconvoluted using Shirley background subtraction and Gaussian–Lorentzian fitting. The surface temperature profiles under Joule heating were monitored using an IR camera (A400, Teledyne FLIR, USA) and a thermocouple thermometer (TA612B, TASI, China). Thermal diffusivity was measured using laser flash analysis (LFA‐467, Netzsch, Germany).

### 4.4 Evaluation of TA Loudspeakers

The VrGO‐ and PrGO‐based TA loudspeakers were fabricated by cutting the films into rectangular pieces (∼ 1 cm × 1.5 cm) and attaching Ag paste/Cu tape electrodes with effective measurement areas of 1 cm × 1 cm. The devices were dried in a desiccator at room temperature for 24 h prior to the measurements. A sinusoidal AC voltage (1–10 V rms, 100 Hz–20 kHz) was applied through the electrodes using a signal generator (SG‐5428A; SIGMA Eltec, South Korea). The emitted sound was detected using a condenser microphone (378B02, PCB Piezotronics, USA) positioned 10 cm from the sample surface, and the signals were analyzed using a dynamic frequency analyzer (National Instruments, USA). All measurements were conducted in a custom‐built anechoic chamber and each experiment was repeated at least three times to ensure reproducibility.

## Conflicts of Interest

The authors declare no conflict of interest.

## Supporting information




**Supporting File 1**: advs74000‐sup‐0001‐SuppMat.docx.

**Supplemental File 2**: advs74000‐sup‐0002‐VideoS1.mov.

## Data Availability

The data that support the findings of this study are available from the corresponding author upon reasonable request.
