# Supplementary material for: Laser‐Architected Shape‐Configurable Vertical Graphene Thermoacoustic Loudspeakers for 3D Acoustic Emission
Source: Adv Sci (Weinh). 2026 Jan 21;13(18):e22911. doi: 10.1002/advs.202522911 (PMC13042591; doi:10.1002/advs.202522911)
Supplement: Supplementary file 1 — Supporting File 1: advs74000‐sup‐0001‐SuppMat.docx. [file ADVS-13-e22911-s001.docx]

Supporting Information

Laser-Architected Shape-Configurable Vertical Graphene Thermoacoustic Loudspeakers for 3D Acoustic Emission

TaeGyeong Lim^1,#^, Se Young Lee^1,#^, Dohyung Lee^1^, Baek Heon Lim^1^, Jeongbo Lee^1^, Wooseok Song^1^, Sun Sook Lee^1^, Sungwoong Park^1^, Jin Kim^2^, Soonmin Yim^1^, Ki-Seok An^1,*^ and Saewon Kang^1,*^

Thin Film Materials Research Center, Korea Research Institute of Chemical Technology, Daejeon, 34114 Republic of Korea

* Corresponding autor. Email: ksan@krict.re.kr (Ki-seok An); skang@krict.re.kr (Saewon Kang);

Department of Materials Science and Engineering, Hanbat National University, Daejeon 34158, Korea

Supplementary note 1.

$R_{1}=\frac{D}{x}$ (S1)

$R_{2}=\frac{D}{y}$ (S2)

where $x$, $y$, and $D$ are the transverse cut spacing, axial cut spacing, and cut length of the Kirigami geometry (Fig. 4a), respectively, and $R_{1}$ and $R_{2}$ are dimensionless parameters.

$\theta={cos}^{-1}\left( \frac{1}{\varepsilon_{A}+1} \right)$ (S3)

$\varepsilon_{r}=\frac{R_{1}-1}{R_{1}+1}\left[ cos\left( {sin}^{-1}\left( \frac{2R_{1}tan\theta}{R_{1}R_{2}-R_{2}} \right) \right)-1 \right]$ (S4)

$\theta_{MAX}={tan}^{-1}\left( \frac{R_{1}R_{2}-R_{2}}{{2R}_{1}} \right)$ (S5)

where $\theta$, $\varepsilon_{A}$, $\varepsilon_{r}$, and $\theta_{MAX}$ are the feature angle, axial strain, transverse strain, and maximum feature angle, respectively (Fig. 4a).


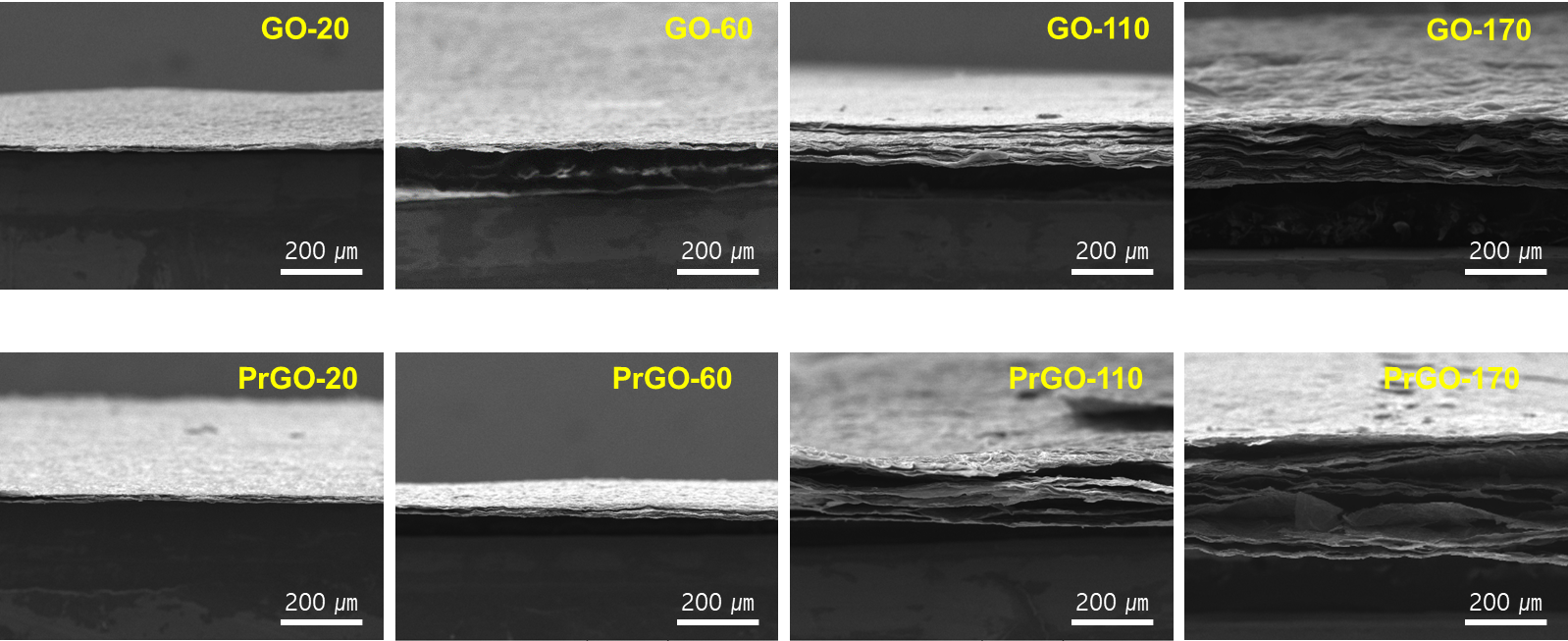


**Increasing film thickness**

Figure S1. Cross-sectional SEM images of GO and PrGO films with different thicknesses.


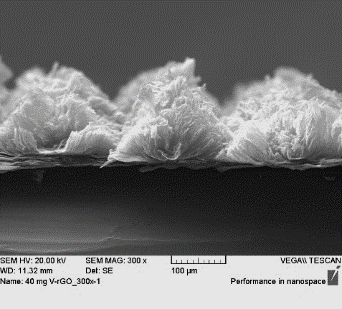


**VrGO-20**


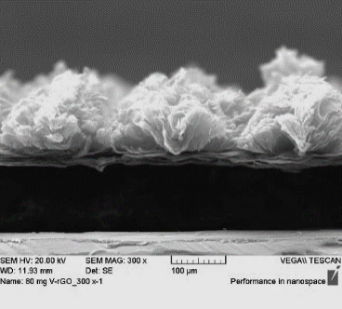


**VrGO-60**


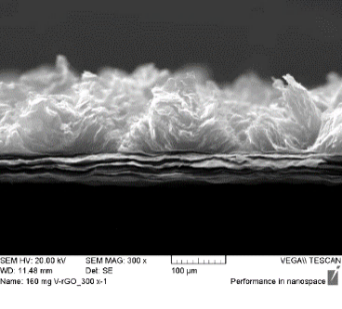


**VrGO-110**


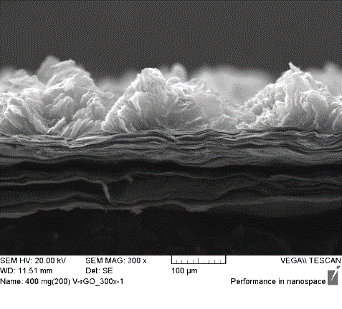


**VrGO-170**

**Increasing film thickness**

Figure S2. Cross-sectional SEM images of VrGO films with different thicknesses.


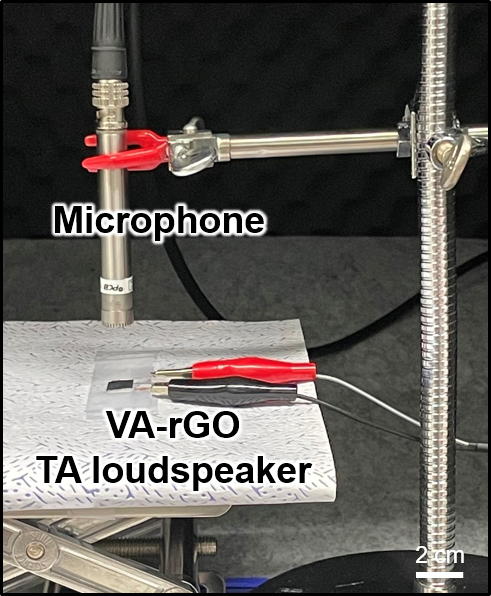


Figure S3. Experimental setup for dynamic frequency-response measurements of TA loudspeakers.

Figure S4. Power spectral density of background noise in the custom-built, lab-scale anechoic chamber.

Figure S5. SPL attenuation of TA loudspeaker as a function of the distance between the microphone and the film.


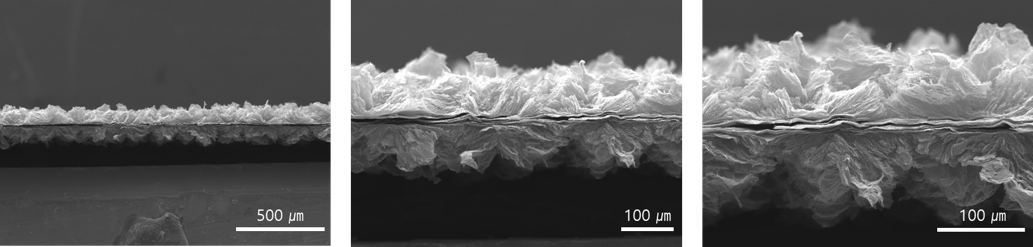


Figure S6. Cross-sectional SEM images of VrGO synthesized by CO_2_ laser scribing on both the front and back sides.


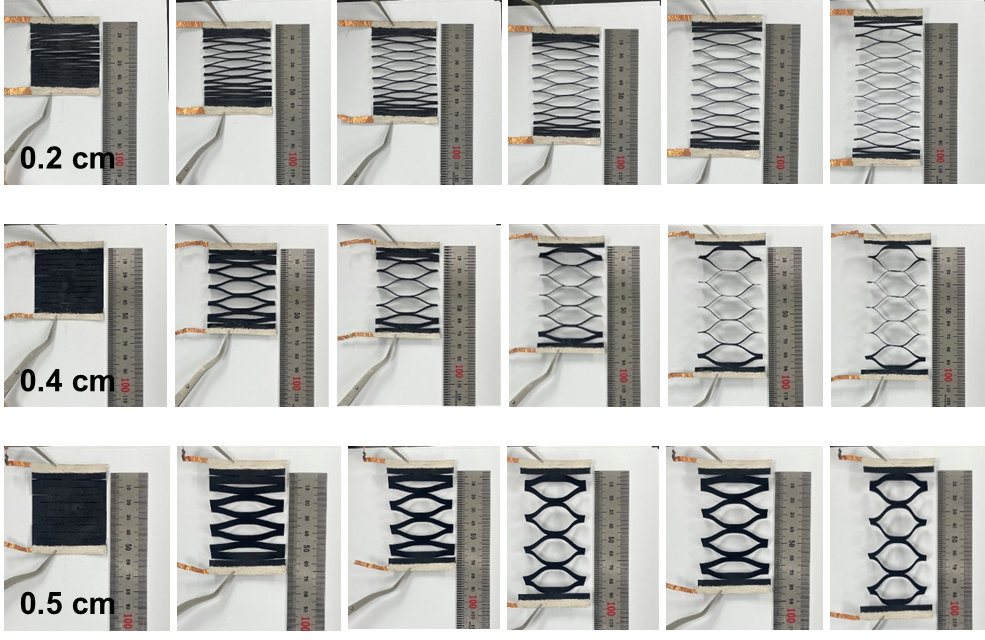


Figure S7. Digital images of kirigami-patterned VrGO TA loudspeakers with different axial cut spacings (y) under various strains of 0, 20, 40, 60, 80, and 100%.


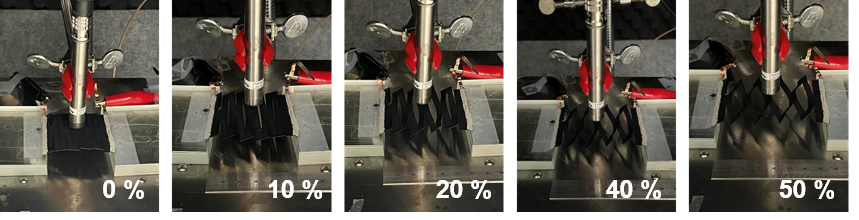


Figure S8. Digital images of SPL measurements for kirigami-patterned VrGO TA loudspeakers with an axial cut spacing of 5 mm under various strains.


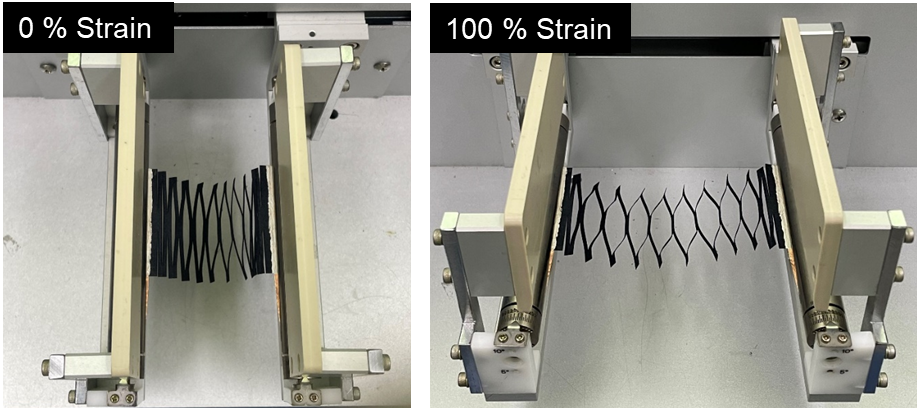


Figure S9. Digital images of kirigami-patterned VrGO TA loudspeaker with an axial cut spacing of 2 mm during durability tests.


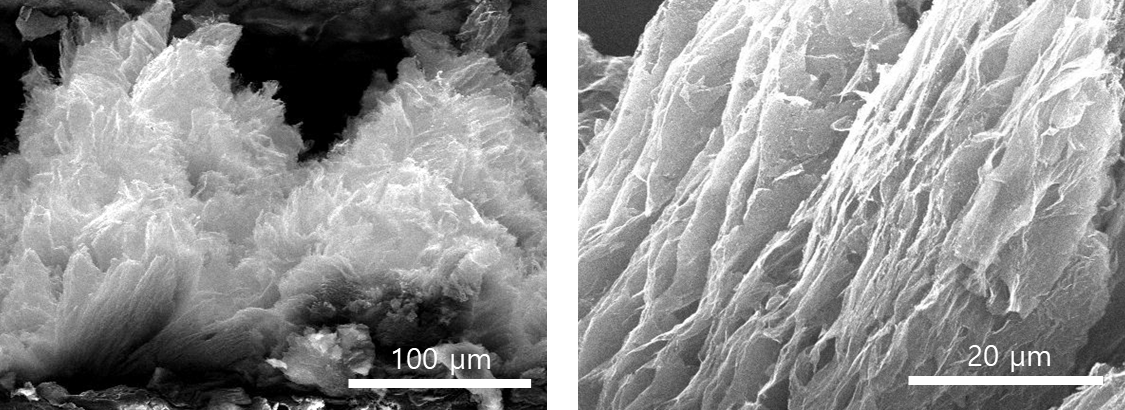


Figure S10. SEM images of VrGO after 1000 cycling tests.


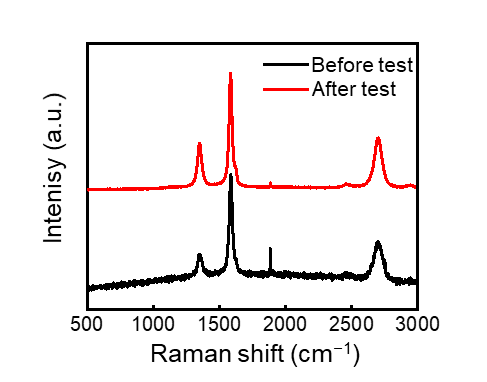


Figure S11. Raman spectra of VrGO before and after 1000 cycling test.


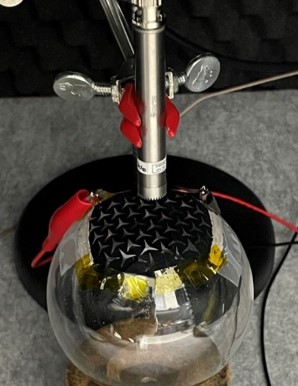


Figure S12. Digital images of SPL measurements for auxetic-patterned VrGO TA loudspeakers.


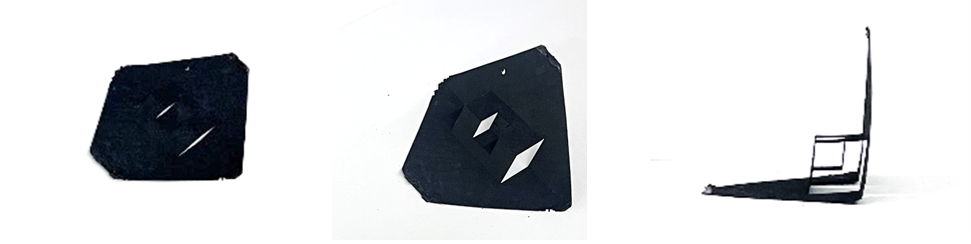


Figure S13. Digital images of pop-up structured VrGO films.


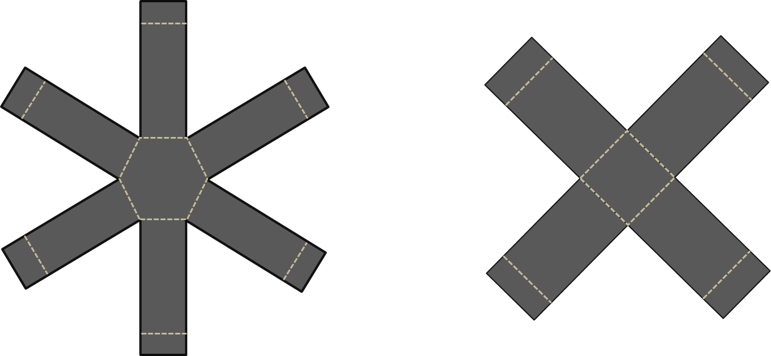


Figure S14. Hexagonal- and rectangular-prismoid-shaped VrGO loudspeaker pattern designs.

Supporting Video

Video S1. Video for thermo-acoustic performance of hexagonal- prismoid VrGO TA loudspeakers in 3D configurations under Joule heating, demonstrating 3D acoustic emission


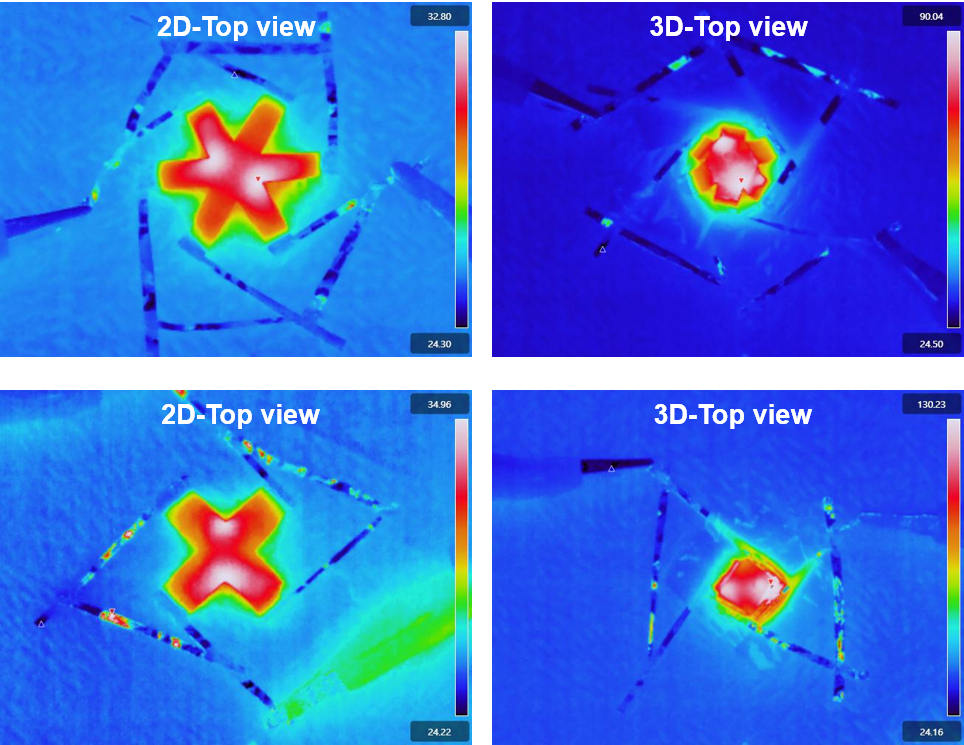


Figure S15. Top-view of IR thermographic images of hexagonal- and rectangular-prismoid-shaped VrGO TA loudspeakers in 2D and 3D configurations under Joule heating.


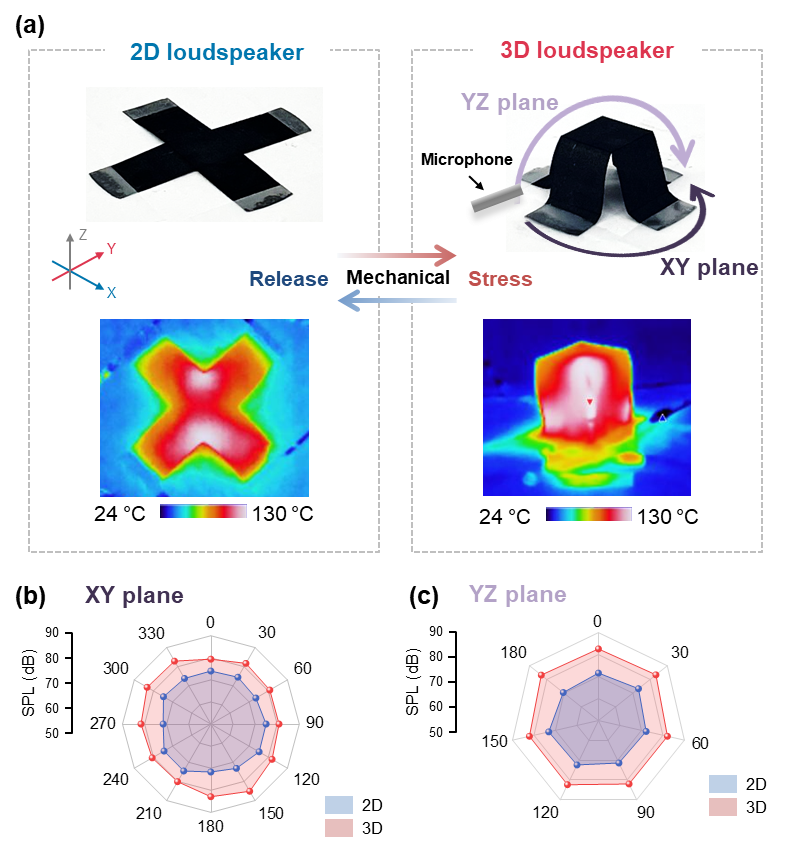


Figure S16. (a) Digital images and IR images of a hexagonal-prismoid-shaped VrGO TA loudspeaker. (b, c) SPL maps of rectangular-prismoid-shaped VrGO loudspeaker in 2D and 3D configurations across the XY and YZ planes.

Table S1. Comparison of TA loudspeakers in terms of materials and performance.

| **TA materials** | **Thickness**  **[μm]** | **Size**  **[cm^2^]** | **SPL at 10 kHz [dB]*** | **Power [W]** | **Frequency range [kHz]** | **Ref. in main text** |
| --- | --- | --- | --- | --- | --- | --- |
| **Multi-layer graphene** | 0.01 | 4 | ****> 50**** | 0.6 | < 14 | 31 |
| **Graphene sheet on paper** | 0.02 | 1 | ****> 70**** | 1** | 0.003-50 | 43 |
| **Single-layer graphene** | 0.00034 | ~ 25 | ****> 80**** | 1** | 0.02-50 | 44 |
| **Free‑standing Graphene** | 0.028 | ~ 2.9 | > 65 | 1 | 1-20 | 45 |
| **N-Doped rGO Aerogel** | 560 | 1 | ****> 75**** | 1 | 1-20 | 46 |
| **prismoids Vertical Graphene** | 3.4 | 1 | ~ 60 | 1.2 | < 17 | 47 |
| **Four-layer CNT** | < 0.1 | ****9**** | ****> 85**** | 12 | 0.1-20 | 48 |
| **SWCNT** | ****0.03**** | ****1**** | > 90 | 1** | 1-100 | 49 |
| **Aluminum Nanowire** | 0.03 | ****0.3**** | ****~ 80**** | 1** | 0.5-20 | 50 |
| **Suspended Metal Wire** | 0.03 | ****17.5**** | ~100 | 1**- | 0.1-40 | 51 |
| **Porous Silicon, nc-Si** | 10 | ****1**** | ****> 70**** | 1 | 5-100 | 52 |
| **PEDOT:PSS** | 0.1 | 2.25 | > 45 | 1** | 1-50 | 53 |
| **This work** | 140.9 | 1 | 85 | ****1**** | ****0.1-20**** |  |

SPL at 10kHz values were estimated from SPL vs frequency curves in literatures

** The input power is reported as a normalized value in literatures
